# Naturally Occurring 2-Methoxy-1,4-naphthoquinone Attenuates Biofilm Formation and Quorum Sensing-Regulated Virulence in *Pseudomonas aeruginosa*

**DOI:** 10.3390/biom16101394

**Published:** 2026-09-24

**Authors:** Rongjia Han, Yuting Bai, Yaxin Zhou, Rongbin Hu, Panpan Li, Bing Li, Yujiao Liang, Jiehang Li, Yubin Bai, Jiyu Zhang

**Affiliations:** 1 Key Laboratory of New Animal Drug Project of Gansu Province, Lanzhou 730050, China; 821012420034@caas.cn (R.H.);; 2 Key Laboratory of Veterinary Pharmaceutical Development, Ministry of Agriculture and Rural Affairs, Lanzhou 730050, China; 3 Lanzhou Institute of Husbandry and Pharmaceutical Sciences, Chinese Academy of Agricultural Sciences, Lanzhou 730050, China; 4 Gansu Institute of Animal Husbandry and Veterinary Sciences, Lanzhou 744000, China

**Keywords:** 2-methoxy-1,4-naphthoquinone, *Pseudomonas aeruginosa*, biofilm, quorum sensing, antibiotic synergy

## Abstract

*Pseudomonas aeruginosa* employs quorum sensing (QS) to coordinate biofilm formation and virulence factor expression, positioning QS as a promising target for anti-infective interventions. This study evaluated the antibiofilm and antivirulence efficacy of 2-methoxy-1,4-naphthoquinone (MNQ), a natural naphthoquinone, against *P. aeruginosa* PA14. At concentrations of 31.25, 62.5, and 125 μg/mL, which had minimal impact on growth, MNQ suppressed biofilm formation (up to 70% at 125 μg/mL), reduced extracellular polymeric substance production, and inhibited quorum sensing-related signalling activity (85.9%), pyocyanin synthesis (78%), as well as swimming and twitching motilities. Notably, MNQ also exhibited antioxidant properties and inhibited efflux pump activity, suggesting a multifaceted mode of action. Mechanistically, qRT-PCR analysis following treatment with MNQ at 125 μg/mL demonstrated significant downregulation of QS regulatory genes (*lasR*, *lasI*, *rhlR*, *rhlI*, *rhlA*) and downstream virulence determinants, corroborating the phenotypic observations. Furthermore, MNQ acted synergistically with azithromycin, cefepime, and ciprofloxacin. Collectively, these findings establish MNQ as a potent QS-directed inhibitor with broad antivirulence activity, underscoring its potential as a lead compound for novel antibiofilm therapeutic strategies.

## 1. Introduction

*Pseudomonas aeruginosa* (*P. aeruginosa*), a Gram-negative opportunistic pathogen, is a remarkably versatile microorganism that has served as a model organism in molecular biology for decades [1,2]. It has been extensively utilised to investigate bacterial gene expression, regulation, intercellular communication (quorum sensing, QS), antibiotic resistance, virulence, and biofilm formation [1]. Owing to its extensive genomic variability, metabolic flexibility, and phenotypic diversity, *P. aeruginosa* exhibits extraordinary functional versatility. These characteristics allow it to thrive in diverse ecological niches. It can act as either a harmless saprophyte or a broad-host-range pathogen [2]. Beyond its environmental ubiquity, *P. aeruginosa* is commonly found in areas associated with human activities and has emerged as a major opportunistic human pathogen, rarely infecting healthy individuals. However, in immunocompromised hosts, it can cause both acute and chronic infections, including those affecting the bloodstream, urinary tract, eyes, and soft tissue wounds [3,4,5]. Pulmonary infections are especially problematic, particularly in patients with cystic fibrosis, chronic lung diseases, or ventilator-associated pneumonia [6,7,8]. Infections caused by *P. aeruginosa* are notoriously difficult to treat due to several factors: its high intrinsic antibiotic tolerance or resistance, the production of numerous intracellular and extracellular virulence factors, its prevalence as a nosocomial pathogen, and its rapid adaptive capacity during infection [9]. Consequently, *P. aeruginosa* has been classified as a serious threat by the Centers for Disease Control and Prevention (CDC) and listed as a critical priority pathogen by the World Health Organisation (WHO) [1,10].

*P. aeruginosa* exhibits resistance to aminoglycosides, fluoroquinolones, and β-lactam antibiotics. The primary mechanisms by which it counteracts antibiotic attack can be categorised into intrinsic, acquired, and adaptive resistance [11,12]. Intrinsic resistance includes low outer membrane permeability [13]. Acquired resistance arises through horizontal gene transfer of resistance determinants or mutational events [9]. Adaptive resistance involves the formation of biofilms, particularly within the lungs of infected patients, where the biofilm serves as a diffusion barrier that restricts antibiotic penetration into bacterial cells [14]. Biofilms are structured aggregates of microbial cells that adhere to each other on biotic or abiotic surfaces and are embedded within a self-produced matrix of extracellular polymeric substances (EPS), including exopolysaccharides, proteins, metabolites, and extracellular DNA (eDNA) [15]. Microbial cells growing within biofilms exhibit significantly reduced susceptibility to antimicrobial agents and host immune responses compared to their planktonic counterparts. Even bacteria lacking intrinsic resistance or protective mutations can become less sensitive to antibiotics when growing in a biofilm state. Given these challenges, there is an urgent need to develop novel antibiofilm compounds targeting these resilient pathogens.

2-methoxy-1,4-naphthoquinone (MNQ) is a naturally occurring 1,4-naphthoquinone derivative produced by plants such as *Impatiens balsamina L*., *Impatiens glandulifera Royle*, and *Swertia calycina*, as well as by the microorganism *Nocardiopsis alba*. Previous studies have demonstrated that MNQ possesses anti-inflammatory, antifungal, antibacterial, and anticancer activities [16]. However, its antibiofilm activity against *P. aeruginosa* remains insufficiently characterised. In the present study, we systematically investigated the inhibitory effects of MNQ on *P. aeruginosa* biofilm formation, explored its underlying mechanisms of action, and evaluated its synergistic potential with conventional antibiotics.

## 2. Materials and Methods

### 2.1. Materials and Bacterial Strains

MNQ (Solarbio Life Sciences, Beijing, China) was dissolved in dimethyl sulfoxide (DMSO) (Solarbio Life Sciences, Beijing, China) to prepare a stock solution of 5000 μg/mL, with the final DMSO concentration in Luria–Bertani (LB) (HuanKai Microbial, Guangzhou, China) medium not exceeding 1%. *P. aeruginosa* PA14 and CV026, a quorum sensing biosensor strain of *Chromobacterium violaceum*, were obtained from the American Type Culture Collection (ATCC). Mueller-Hinton broth (MHB) (HuanKai Microbial, Guangzhou, China), LB broth (HuanKai Microbial, Guangzhou, China), and LB agar (HuanKai Microbial, Guangzhou, China) were used for bacterial culture. *P. aeruginosa* PA14 was routinely cultured in LB medium. Unless otherwise specified, MNQ was used at final concentrations of 31.25, 62.5, and 125 μg/mL throughout the experiments.

### 2.2. Growth Curve and Metabolic Activity

To evaluate bacterial growth, MNQ was added to the bacterial suspension at the concentrations specified in Section 2.1. Equal volumes of the bacterial suspension were inoculated into 96-well (Corning Costar^®^ 3599, Corning, NY, USA) plates along with designated control groups. The plates were incubated at 37 °C, and the optical density at 600 nm (OD_600_) was recorded at regular intervals to construct growth curves.

In parallel, the metabolic activity of *P. aeruginosa* was assessed using the Alamar Blue (AB) assay, as previously described by Swetha et al., 2019 [17]. Briefly, bacterial cells from each well were collected into sterile centrifuge tubes, pelleted by centrifugation at 2947× *g* for 10 min, washed twice with phosphate-buffered saline (PBS, pH 7.2), and finally resuspended in 1 mL of PBS (pH 7.2). Then, 10 μL of AB dye (Invitrogen, Thermo Fisher Scientific, Waltham, MA, USA) was added to each tube, and the mixtures were incubated in the dark at 37 °C for 1 h. A blank control containing culture medium, MNQ, and AB but no bacteria was included. The metabolic activity was calculated from the absorbance readings at 570 nm and 600 nm using Equation (1). All experiments were performed in three independent replicates as mean ± standard deviation (SD).

(1) $$\mathrm{Metabolic}\;\mathrm{activity}\;(\%)=\frac{\left((Eoxi(OD600)\times T(OD570)-(Eoxi\left(OD570\right)\times T(OD600)\right)}{\left((Ered(OD570)\times B(OD600)-(Ered\left(OD600\right)\times B(OD570)\right)}\times 100\%$$

Eoxi: Molar extinction coefficient of oxidised Alamar Blue (AB);

Ered: Molar extinction coefficient of reduced Alamar Blue (AB);

T: Absorbance value of the test sample containing bacteria, MNQ, and AB;

B: Absorbance value of the blank control containing culture medium, MNQ, and AB but no bacteria.

### 2.3. Swarming, Swimming, and Twitching Motility Assay

Swarming, swimming, and twitching motilities were assayed as previously described by Liu et al., 2020 [18], with minor modifications.

The effects of MNQ on the swimming, swarming, and twitching motilities of *P. aeruginosa* PA14 were evaluated using semi-solid agar plates with slight modifications to the medium composition and agar concentration. For all assays, MNQ was incorporated into the medium at the concentrations specified in Section 2.1, while plates without MNQ served as controls. The plates were prepared in a single layer and allowed to dry at room temperature for 1 h. Subsequently, 1 μL of PA14 culture (OD_600_ = 1.0) was inoculated onto the centre of each plate, followed by incubation at 37 °C for 16 h.

For the swarming motility assay, 20 mL of LB medium containing 0.5% (*w*/*v*) bacto agar, 0.5% (*w*/*v*) casamino acids, and 0.5% (*w*/*v*) glucose was used. For the swimming motility assay, the same medium composition was used except that the bacto agar concentration was reduced to 0.3% (*w*/*v*). For the twitching motility assay, 20 mL of LB medium containing 1% (*w*/*v*) bacto agar, 0.5% (*w*/*v*) casamino acids, and 0.5% (*w*/*v*) glucose was used. Following incubation, the diameter of the bacterial migration zone was measured to quantify motility.

### 2.4. Biofilm Inhibition and Elimination

The antibiofilm activity of MNQ was determined by the crystal violet (CV) staining method as previously described by Pejcic et al., 2020 [19]. In brief, a suspension of *P. aeruginosa* PA14 (OD_600_ = 0.01) was added to 96-well plates (Corning Costar^®^ 3599, Corning, NY, USA) containing TSB medium. MNQ was added at the concentrations specified in Section 2.1. The plates were then incubated at 30 °C for 24 h. Following incubation, each well was gently washed three times with PBS to eliminate non-adherent bacteria. The attached biofilms were subsequently fixed with formaldehyde (20 min), stained with 0.1% crystal violet (30 min), and thoroughly rinsed. The crystal violet retained within the biofilms was extracted using 95% ethanol, and the absorbance was read at 595 nm on a Multiskan Go Reader (Thermo Fisher Scientific, Waltham, MA, USA). For biofilm elimination, MNQ was added 24 h after biofilm formation at the concentrations specified in Section 2.1, followed by incubation for an additional 24 h. All other operations were the same as those described in the biofilm inhibition assay section. Biofilm inhibition and elimination were calculated as follows:

(2) $$\text{Biofilm}\;\text{inhibition}/\text{elimination}\;(\%)\;=\;\frac{ODX-ODY}{ODX-ODZ}\times 100\%$$

where (ODX) represents the absorbance of the positive control containing bacteria without MNQ treatment, (ODY) represents the absorbance of the MNQ-treated group, and (ODZ) represents the absorbance of the negative control containing the same medium and reagents but without bacteria or MNQ.

### 2.5. EPS

EPS production was quantitatively estimated using ruthenium red staining as previously described by Nair et al., 2018 [20]. Briefly, PA14 bacterial suspension (OD_600_ = 0.01) was mixed with different concentrations of MNQ in TSB medium and added to a 96-well plate. The untreated PA14 bacterial suspension without MNQ was used as the positive control (AP). A blank control containing only ruthenium red solution without bacterial suspension was included to determine the background absorbance. The plate was incubated statically at 30 °C for 24 h. After washing with PBS, 0.01% ruthenium red solution (Yuanye, Shanghai, China) was added to each well. The plate was incubated at 37 °C for 1 h. The residual staining solution was then transferred to a new 96-well plate, and the absorbance was measured at 450 nm using a Multiskan Go Reader.

EPS inhibition was calculated as follows [20]:

(3) $$\text{EPS}\;\text{inhibition}\;(\%)\;=\;\frac{AS-AP}{AB-AP}\times 100\%$$

where:

AB: absorbance of the blank;

AS: absorbance of the sample;

AP: absorbance of the untreated PA14 bacterial suspension without MNQ (positive control).

### 2.6. Pyocyanin and Quorum Sensing Assay

Overnight cultures of *P. aeruginosa* strains were adjusted to an optical density of 0.5 at 590 nm (OD_590_ = 0.5) and then diluted 1:50 in 10 mL of LB broth with or without MNQ at the concentrations specified in Section 2.1. Following 24 h of incubation at 37 °C, the culture supernatants were collected and filter sterilised. For pyocyanin quantification, 3 mL of chloroform was added to 5 mL of the cell-free supernatant and vortexed. After centrifugation (2947× *g*, 5 min), the chloroform layer was recovered and mixed with 1 mL of 0.2 M HCl. The mixture was vortexed and centrifuged, and the absorbance of the HCl phase containing pyocyanin was measured at 520 nm using an EnVision Multilabel Reader (Perkin Elmer, Waltham, MA, USA), as previously described by Kong et al., 2005 [21]. To account for possible interference of MNQ with absorbance at 520 nm, MNQ-only extraction blanks (without bacteria) were prepared in parallel at the same concentrations and processed identically. Absorbance values were corrected by subtracting the corresponding MNQ blank readings. A blank control consisting of 0.2 M HCl was used for baseline correction.

Pyocyanin inhibition was calculated as follows:

(4) $$\text{Pyocyanin}\;\text{inhibition}\;(\%)\;=\;(1\;-\;\frac{AS-AB}{AC})\times 100\%$$

where AS is the absorbance of the sample at 520 nm, AB is the absorbance of the corresponding MNQ-only extraction blank at 520 nm, and AC is the blank-subtracted absorbance of the untreated PA14 control at 520 nm.

For the quorum sensing assay, PA14 cultures were incubated overnight at 37 °C with MNQ at the concentrations specified in Section 2.1 under shaking conditions, according to the method described by Martinelli et al., 2004 [22], with minor modifications. The cultures were centrifuged at 11,788× *g* for 8 min at room temperature, and the supernatants were collected and filter sterilised through 0.22 μm membrane filters. The resulting cell-free supernatants were used for the quorum sensing assay. A single colony of *Chromobacterium violaceum* CV026 was inoculated into LB broth and cultured at 30 °C with shaking at 150 rpm for 16 h. The CV026 culture was added to LB agar supplemented with C6-HSL at a final concentration of 10 μM and mixed thoroughly before pouring the plates. After solidification, wells were punched using a sterile 1 mL pipette tip, and 100 μL of each PA14 culture supernatant was added to the corresponding well. The supernatant from untreated PA14 cultures was used as the control, while an MNQ-only control was included to exclude the direct effect of MNQ on CV026. The MNQ-only control was not included in the calculation of quorum sensing inhibition. The plates were incubated at 30 °C for 24 h. Quorum sensing inhibition was evaluated by observing the reduction in violacein production and measuring the diameter of the violacein inhibition zone. Three independent replicates were performed for each group.

QS inhibition was calculated as follows:

(5) $$QS\;\text{inhibition}\;(\%)\;=\;(\frac{DS-DC}{DC})\times 100\%$$

where DS is the diameter of the violacein inhibition zone in the MNQ-treated group, and DC is the diameter of the violacein inhibition zone in the untreated PA14 control group. The MNQ-only control was included to exclude the direct effect of MNQ on CV026 and was not included in the calculation.

### 2.7. Outer Membrane Permeability Assay, Cell Membrane Integrity Assay, Reactive Oxygen Species Detection and EtBr Efflux Assay

The bacterial strain PA14 was cultured overnight at 37 °C with shaking at 200 rpm. The bacterial cells were subsequently washed and resuspended in 5 mM HEPES buffer (pH 7.0) supplemented with 5 mM glucose. The bacterial suspension was adjusted to an OD_600_ of 0.3 using the same buffer, followed by the addition of the fluorescent dye. After incubation at 37 °C for 30 min, 1 mL of the bacterial suspension was mixed with MNQ at the concentrations specified in Section 2.1, together with untreated and positive control groups (TritonX-100 was used as the positive control for the NPN uptake and PI staining assays, Rosup was used as the positive control for the intracellular ROS assay, and 100 μM CCCP was used as the positive control for the efflux pump activity assay). After incubation for 1 h, 200 μL of each bacterial suspension was transferred to a transparent, flat-bottom 96-well microplate (Zhenxuan, Beijing, China). Fluorescence intensity or luminescence signals were subsequently measured using a microplate reader (Thermo Scientific, Waltham, MA, USA).

N-phenyl-1-naphthylamine (NPN, 10 µM, Beyotime, Shanghai, China) uptake was monitored at excitation/emission of 350/420 nm to evaluate membrane permeabilisation, while propidium iodide (PI, 0.5 mM, Beyotime, Shanghai, China) stainin-g was measured at an excitation/emission wavelength of 535/615 nm to assess membrane integrity; TritonX-100 (Beyotime, Shanghai, China) was used as a positive control, as previously described by Zhang et al., 2026 [23].

Intracellular reactive oxygen species (ROS) levels were detected using 2′,7′-dichlorodihydrofluorescein diacetate (DCFH-DA, 10 µM, Beyotime, Shanghai, China). Briefly, the bacterial suspension was incubated with DCFH-DA at 37 °C for 30 min, followed by treatment with MNQ at the concentrations specified in Section 2.1. After incubation for 1 h at 37 °C, fluorescence intensity was measured at an excitation wavelength of 488 nm and an emission wavelength of 525 nm. Untreated bacteria were used as the negative control, and Rosup was used as a positive control, as previously described by Yi et al., 2025 [24].

For efflux pump activity, bacterial suspensions were washed three times with 10 mM HEPES buffer (Beyotime, Shanghai, China), adjusted to OD_600_ = 0.3, and pre-incubated with 5 µM ethidium bromide (EtBr, Beyotime, Shanghai, China) for 30 min at 37 °C. One millilitre aliquots were then treated with graded concentrations of MNQ or with 100 µM carbonyl cyanide 3-chlorophenylhydrazone (CCCP) as a reference. After 1 h of incubation at 37 °C, EtBr fluorescence was measured at excitation/emission of 525/600 nm using a Varioskan Flash luminometer (Thermo Scientific, Waltham, MA, USA), as previously described by Han et al., 2023 [25].

### 2.8. qRT-PCR

To investigate the effect of MNQ on the transcriptional expression of biofilm-related genes in *P. aeruginosa*, quantitative real-time PCR (qRT-PCR) was performed as previously described by Bai et al., 2025 [26]. Bacterial cells were cultured in the presence or absence of MNQ (125 μg/mL) at 37 °C for 24 h. Total RNA was extracted using a Bacterial RNA Kit (Omega Bio-tek, Norcross, GA, USA), and its concentration was determined with a NanoDrop OneC spectrophotometer (Thermo Scientific, Madison, WI, USA), while integrity was confirmed by agarose gel electrophoresis. Complementary DNA (cDNA) was then synthesised from the extracted RNA using the PrimeScript™ RT Reagent Kit with gDNA Eraser (Takara Bio, Kusatsu, Japan). The qRT-PCR reaction was conducted with TB Green^®^ Premix Ex Taq™ II (Tli RNaseH Plus) (Takara Bio, Kusatsu, Japan) on a QuantStudio 6 Flex Real-Time PCR System (Applied Biosystems) using QuantStudio Real-Time PCR Software version 1.7.2. The 16S rRNA gene was used as the internal control, and relative expression levels were calculated using the 2^−ΔΔCt^ method. The thermal cycling protocol consisted of an initial denaturation at 95 °C for 30 s, followed by 40 cycles of 95 °C for 5 s and 60 °C for 34 s. Three biological replicates were performed for each condition. All primers employed in this study are listed in Supplementary Table S1.

### 2.9. Antibacterial Activity of MNQ in Combination with Antibiotics

To further evaluate the antibacterial activity of MNQ in combination with antibiotics, the MICs of MNQ and the tested antibiotics were first determined using a broth microdilution method, as previously described by Liu et al., 2022 [27]. Briefly, bacteria in the exponential growth phase were diluted 1:1000 in MHB to obtain a final bacterial concentration of approximately 5 × 10^5^ CFU/mL. The bacterial suspensions were mixed with two-fold serial dilutions of MNQ (0.061 – 500 μg/mL) or the tested antibiotics in sterile 96-well plates and incubated at 37 °C for 16–18 h. The MIC was defined as the lowest concentration that completely inhibited visible bacterial growth.

The antibacterial efficacy of MNQ was evaluated according to the method described by Liu et al., 2022 [27]. Briefly, overnight bacterial cultures were diluted to an OD_600_ of 0.01 and subsequently mixed with various concentrations of antibiotics (1/2 MIC, 1/4 MIC, and 1/8 MIC) in the presence (125 μg/mL) or absence of MNQ. The mixtures were then incubated at 37 °C for 16–18 h. After incubation, bacterial growth was evaluated by measuring the OD_600_ using a microplate reader. The antibacterial effect of the treatments was assessed by comparing the OD_600_ values with those of the untreated control. All assays were conducted in three independent replicates.

Checkerboard assays were subsequently performed to determine the interaction between MNQ and each antibiotic. Two-fold serial dilutions of MNQ and antibiotics were prepared in an 8 × 8 matrix, followed by inoculation with bacterial suspensions at a final concentration of 5 × 10^5^ CFU/mL. After incubation at 37 °C for 18 h, bacterial growth in each well was assessed by measuring the OD_600_ using a microplate reader. The fractional inhibitory concentration (FIC) of each compound was calculated as the MIC of the compound in combination divided by its MIC alone. The fractional inhibitory concentration index (FICI) was calculated according to the following equation:

(6) $$FICI\;=\;{FIC}_{MNQ}+{FIC}_{\text{antibiotic}}=\;\frac{MIC(MNQ\;combination)}{MIC(MNQ\;alone)}+\frac{MIC(antibiotic\;combination)}{MIC(antibiotic\;alone)}$$

where FIC_MNQ_ is the fractional inhibitory concentration of MNQ, calculated as the MIC of MNQ in combination with the antibiotic MIC (MNQ combination) divided by the MIC of MNQ alone MIC(MNQ alone). FIC_antibiotic_ is the fractional inhibitory concentration of the antibiotic, calculated as the MIC of the antibiotic in combination with MNQ MIC(antibiotic combination) divided by the MIC of the antibiotic alone MIC(antibiotic alone).

FICI ≤ 0.5 was interpreted as synergistic activity, 0.5 < FICI ≤ 1.0 indicated an additive effect, and FICI > 1.0 indicated an indifferent effect, as previously described by Xia et al., 2024 [28]. All assays were conducted in three independent replicates.

### 2.10. Statistical Analysis

The results were analysed by GraphPad Prism 8.0.2 software. Data were expressed as means ± SD. Statistical significance was assessed using one-way ANOVA followed by Tukey’s or Dunnett’s multiple comparisons test, or two-way ANOVA followed by Tukey’s or Šídák’s multiple comparisons test, as appropriate. A *p* value <0.05 was considered significant.

### 2.11. Use of Artificial Intelligence

During the preparation of this manuscript, ChatGPT 5.5 was used for grammar correction, sentence structure improvement, and general language polishing. The authors have carefully reviewed and edited all content and take full responsibility for the final manuscript.

## 3. Results

### 3.1. Effects of MNQ on Growth and Metabolic Activity

To evaluate the impact of MNQ on *P. aeruginosa* PA14, we assessed bacterial growth (via optical density at 600 nm, OD_600_) and metabolic activity under varying concentrations of MNQ (31.25, 62.5, and 125 μg/mL). As shown in Figure 1A, MNQ treatment resulted in a modest reduction in OD_600_ values compared with the untreated control, with significant differences observed between the MNQ-treated groups and the untreated control, except for the comparison between 31.25 and 62.5 μg/mL MNQ (*p* = 0.4322). Similarly, metabolic activity of PA14, expressed as a percentage of the untreated control, showed a decreasing trend with increasing MNQ concentrations (Figure 1B); however, the differences among the MNQ-treated groups were not statistically significant. Specifically, the metabolic activity decreased significantly at 62.5 (93.89%) and 125 μg/mL (92.96%) compared to the untreated control (*p* = 0.0172 and *p* = 0.0078, respectively), while the 31.25 μg/mL group (97.79%) showed only a slight reduction, which did not reach statistical significance (*p* = 0.5140).

### 3.2. Evaluation of the Effects of MNQ on P. aeruginosa Motility

*P. aeruginosa* employs swarming, swimming, and twitching motilities as key virulence attributes essential for its pathogenesis and infection establishment. Here, we investigated the impact of MNQ on these three motile behaviours of *P. aeruginosa*. As shown in Figure S1A–F, MNQ significantly inhibited twitching (Figure S1A,D) and swimming (Figure S1B,E) motilities of *P. aeruginosa*, with progressively reduced migration distances observed at increasing MNQ concentrations (125, 62.5, and 31.25 μg/mL) compared with the negative control. In contrast, MNQ enhanced swarming motility of *P. aeruginosa* compared with the control group, with the most pronounced enhancement observed at 62.5 μg/mL (Figure S1C,F).

### 3.3. Disruption of Biofilm Development by MNQ

To assess the antibiofilm potential of MNQ, we evaluated its effects on both biofilm formation and preformed biofilm eradication in *P. aeruginosa* PA14 using a crystal violet assay across varying concentrations (31.25, 62.5, 125 μg/mL). As shown in Figure 2A, MNQ treatment reduced biofilm biomass during the biofilm inhibition assay, with inhibition rates reaching approximately 70% at 125 μg/mL, 64% at 62.5 μg/mL, and 60% at 31.25 μg/mL. MNQ treatment reduced biofilm biomass at all tested concentrations; however, the differences among the MNQ-treated groups were not statistically significant. In parallel, the eradication assay revealed that MNQ also disrupted established biofilms, achieving eradication rates of roughly 51% at 125 μg/mL, 45% at 62.5 μg/mL, and 41% at 31.25 μg/mL (Figure 2B). Collectively, these findings indicate that MNQ not only prevents biofilm development likely by interfering with early-stage adhesion, microcolony formation, or extracellular polymeric substance (EPS) deposition but also possesses the capacity to dismantle pre-existing biofilms, highlighting its dual functionality as both a prophylactic and a therapeutic antibiofilm agent against PA14.

### 3.4. Effects of MNQ on Extracellular Polymeric Substance (EPS) Production in P. aeruginosa

Extracellular polymeric substances (EPS) are critical structural components of bacterial biofilms, contributing to biofilm stability, antibiotic tolerance, and immune evasion. To determine whether MNQ disrupts biofilm integrity by modulating EPS production, we evaluated the effects of MNQ on EPS production at different concentrations (31.25, 62.5, and 125 μg/mL). As shown in Figure 3, MNQ markedly inhibited EPS production at all tested concentrations. At 31.25 μg/mL, MNQ reduced EPS production by approximately 47.4% relative to the untreated control (*p* < 0.0001). Notably, a two-fold increase in concentration (62.5 μg/mL) enhanced EPS inhibition to 77.4%, representing a 1.63-fold increase in efficacy compared to the 31.25 μg/mL group. Further escalation to 125 μg/mL yielded 78% inhibition, suggesting a plateau in EPS suppression at higher doses.

### 3.5. Effects of MNQ on Pyocyanin Production and Quorum Sensing-Related Signalling Activity in P. aeruginosa

As a key virulence factor, pyocyanin is a redox-active phenazine pigment secreted by *P. aeruginosa* that facilitates bacterial colonisation, disrupts host cell redox balance, and promotes tissue damage. To further elucidate the antivirulence potential of MNQ, we assessed its inhibitory effect on pyocyanin production at varying concentrations (31.25, 62.5, and 125 μg/mL).

MNQ markedly reduced pyocyanin production at all three concentrations, with inhibition rates of 92.7%, 97.9%, and 83.5%, respectively (Figure 4A). Although substantial inhibition was observed at all tested concentrations, the effect was not strictly concentration dependent, as the inhibition rate decreased from 97.9% at 62.5 μg/mL to 83.5% at 125 μg/mL. As shown in Figure 4B, MNQ also inhibited violacein production by *Chromobacterium violaceum* CV026 in a concentration-dependent manner, with inhibition rates of 16.7%, 52.6%, and 85.9% at 31.25, 62.5, and 125 μg/mL, respectively. Exogenous C6-HSL partially restored violacein production, supporting the inhibitory effect of MNQ on AHL-dependent quorum-sensing signalling.

### 3.6. Effects of MNQ on ROS Accumulation, Efflux Pump Activity, and Membrane Integrity

As shown in Figure 5, the effects of MNQ on intracellular ROS accumulation, efflux pump activity, and membrane integrity in *P. aeruginosa* PA14 were evaluated using DCFH-DA, EtBr, NPN, and PI fluorescence probes, respectively. For ROS production (Figure 5A), MNQ treatment resulted in a decreasing trend in fluorescence intensity with increasing concentrations. Efflux pump activity assessed by EtBr accumulation (Figure 5B) showed increased fluorescence intensity following MNQ treatment (Compare with the control group), suggesting reduced EtBr efflux and inhibition of efflux pump activity. The NPN fluorescence peak was significantly reduced, and the permeability of the outer membrane decreased (Compare with the control group) (Figure 5C). In contrast, PI staining (Figure 5D) showed significant differences between the MNQ-treated groups and the untreated control, but PI fluorescence intensity decreased following MNQ treatment rather than increased, indicating no increase in membrane permeability under the tested conditions. Collectively, these results suggest that MNQ reduces intracellular ROS accumulation and may inhibit efflux pump activity at the tested concentrations, without causing detectable membrane damage.

### 3.7. Evaluation of Biofilm-Related Gene Expression

To further elucidate the molecular mechanism by which MNQ disrupts *P. aeruginosa* biofilm formation, we employed quantitative real-time PCR (qRT-PCR) to analyse the expression of quorum sensing (QS)-controlled virulence genes, biofilm-associated genes, and related regulatory elements. As shown in Figure 6, treatment with MNQ (at a single, optimised concentration of 125 μg/mL) markedly altered the transcriptional landscape of these genes. The expression of core QS system genes including *pqsR*, *lasR*, *lasI*, *lasB*, *rhlA*, *rhlI* and *rhlR* was significantly downregulated, indicating that MNQ suppresses both the *las* and *rhl* QS cascades. In contrast, the biofilm development regulators *dipA* and *bdlA* were notably upregulated, potentially reflecting a compensatory stress response. These transcriptional changes collectively suggest that MNQ disrupts biofilm formation by interfering with QS signalling and downstream gene expression, thereby impairing the coordinated production of biofilm matrix components and virulence factors.

### 3.8. Combined Effects of MNQ with Antibiotics Against PA14

The emergence of antibiotic resistance in *P. aeruginosa* urgently necessitates the development of novel antimicrobial adjuvants to potentiate antibiotic efficacy, and targeting bacterial biofilms represents a promising alternative strategy as it acts on biofilm structural integrity rather than pathogen viability, thereby exerting weaker selective pressure for resistance compared to conventional antibiotics. Here, we investigated the combined effects of MNQ with five clinically relevant antibiotics azithromycin, cefepime, ciprofloxacin, tetracycline, and polymyxin B, against PA14. MNQ exhibited an MIC of >500 μg/mL against PA14, indicating negligible direct antibacterial activity at the concentrations investigated (≤125 μg/mL) (Table 1). MBC was therefore not determined, as the study focused on the antibiofilm and antivirulence effects of MNQ rather than its bactericidal activity. In combination with MNQ, the MICs of azithromycin and cefepime decreased from 125 to 31.25 μg/mL and from 3.9 to 0.977 μg/mL, respectively, with FICI values falling within the synergistic range (Table 1). Because the MIC of MNQ was >500 μg/mL, the corresponding FICI values could not be determined as exact numerical values and were therefore expressed as ranges. For ciprofloxacin, the MIC decreased from 0.122 to 0.061 μg/mL, corresponding to an additive interaction based on the FICI criterion. In contrast, MNQ did not further reduce the MICs of tetracycline or polymyxin B, suggesting that its adjuvant effect was antibiotic dependent. Consistent with these MIC findings, co-treatment with MNQ and azithromycin or cefepime at 1/2 MIC markedly reduced PA14 viability compared with the corresponding antibiotic monotherapies (Figure 7A,B). The reduction in bacterial viability increased with increasing concentrations of the combinations, indicating a concentration-dependent enhancement of antibiotic activity. MNQ also enhanced the bacteriostatic activity of ciprofloxacin at 1/4 and 1/8 MIC (Figure 7C). However, this effect was not dose dependent, and the combination at 1/2 MIC was less effective than ciprofloxacin monotherapy. In contrast, MNQ failed to enhance the antibacterial activity of tetracycline or polymyxin B, as bacterial viability in the combination groups was comparable to or higher than that observed with the corresponding antibiotic monotherapies across the tested concentrations (Figure 7D,E). Collectively, these findings demonstrate that MNQ possesses weak intrinsic antibacterial activity but can potentiate the activity of selected antibiotics, particularly azithromycin and cefepime, against *P. aeruginosa* PA14.

## 4. Discussion

A critical clinical challenge posed by biofilms is their intrinsic tolerance to antimicrobial agents and host immune clearance, a phenomenon that occurs even in bacteria lacking specific resistance genes or protective mutations [29]. Beyond antibiotic tolerance, biofilm-embedded cells also exhibit heightened resilience to environmental stressors, including ultraviolet radiation, metal toxicity, acid exposure, desiccation, and pH fluctuations [30]. Consequently, the identification of novel antibiofilm agents has become an urgent priority in infectious disease research. Previous studies have shown that MNQ enhances the antibacterial activity of tetracycline against *Escherichia coli* J53p47EC and *Klebsiella pneumoniae* K12016p47EC carrying tet(X4). MNQ may also interfere with bacterial efflux pump activity [31]. These findings, together with its previously reported antibiofilm potential, suggest that MNQ may exert antibacterial effects beyond direct growth inhibition. Importantly, MNQ showed negligible cytotoxicity toward Caco-2, HEK-293, HeLa, and human HaCaT keratinocytes, supporting its potential suitability for further antimicrobial development [31,32]. Here, we show that MNQ inhibits biofilm formation and several associated virulence phenotypes in *P. aeruginosa* PA14. Notably, these effects coincided with changes in bacterial metabolic activity at 62.5 and 125 μg/mL MNQ, indicating that growth-related effects may contribute to the observed antibiofilm and antivirulence activities. Thus, MNQ may have potential for attenuating *P. aeruginosa* pathogenicity, although its effects should be interpreted in the context of bacterial growth.

In this study, we demonstrate that the natural quinone MNQ inhibits and disrupts biofilms formed by the hypervirulent *P. aeruginosa* strain PA14. These effects were accompanied by reduced EPS production and pyocyanin synthesis, supporting a broad effect of MNQ on virulence-associated phenotypes. *P. aeruginosa* is capable of producing at least three major EPS components: alginate, polysaccharide synthesis locus (Psl)-associated polysaccharide, and pellicle (Pel)-associated polysaccharide, each of which may be either cell-associated or released into the surrounding environment [1]. Although individual strains typically favour one of these polymers, each contributes distinctly to biofilm architecture, mediating cell–cell interactions and imparting unique biophysical properties to the matrix. The PA14 strain specifically produces Pel, a cationic, partially de-N-acetylated linear polymer of α-1,4-N-acetylgalactosamine, composed primarily of repeating dimers of galactosamine and N-acetylgalactosamine [1]. Pel has been shown to confer protection against aminoglycoside antibiotics and to interact with eDNA [33], thereby providing structural stability to the biofilm matrix. Given that MNQ treatment reduced EPS production, it is plausible that MNQ interferes with Pel-dependent matrix integrity; however, the precise molecular targets warrant further investigation.

Pyocyanin, a redox-active virulence factor responsible for the characteristic blue-green pigmentation of *P. aeruginosa*, is known to generate reactive oxygen species (ROS) and induce oxidative stress in host cells [34]. Interestingly, MNQ inhibited pyocyanin production at all tested concentrations, with the greatest inhibition observed at 62.5 μg/mL rather than 125 μg/mL. This non-linear response may be partly due to reduced bacterial metabolic activity at 125 μg/mL MNQ, which may alter pyocyanin biosynthesis (Figure 1B). In contrast, MNQ exhibited a clear concentration-dependent inhibitory effect on QS-associated violacein production in the CV026 biosensor assay. The partial restoration of violacein production by exogenous C6-HSL further supports the interference of MNQ with AHL-dependent QS-associated signalling, although the precise molecular target of MNQ within this regulatory network remains to be determined. MNQ treatment not only suppressed pyocyanin production but also resulted in a decreasing trend in overall ROS levels with increasing concentrations (Figure 5A), suggesting that MNQ possesses intrinsic antioxidant properties that may counteract the pro-oxidant activity of pyocyanin. The RND-type efflux pumps MexAB-OprM, MexCD-OprJ, MexEF-OprN, and MexXY-OprM are well-established contributors to intrinsic and acquired resistance in *P. aeruginosa*, mediating the extrusion of β-lactams, quinolones, and aminoglycosides [9]. MNQ reduced efflux pump activity at all tested concentrations, suggesting that MNQ may interfere with the efflux-mediated resistance phenotype of *P. aeruginosa*. This finding is consistent with the study by Xu et al., which also reported attenuation of efflux pump activity following treatment with MNQ [31]. Although the underlying mechanism remains unclear, the observed reduction in efflux activity with increasing MNQ concentrations suggests that MNQ may act as a potential efflux pump inhibitor, thereby contributing to its antibiofilm and antivirulence effects. Using the NPN uptake assay, we observed a dose-dependent decrease in NPN fluorescence intensity, indicative of reduced outer membrane permeability. Meanwhile, PI staining showed significant differences among the MNQ-treated groups, except between 31.25 and 125 μg/mL. These results collectively demonstrate that MNQ does not function as a conventional membrane-disrupting agent. This is consistent with the inherently low outer membrane permeability of *P. aeruginosa*, which is approximately 12- to 100-fold lower than that of *Escherichia coli* [35]. Therefore, the modest antibacterial activity of MNQ is unlikely to be attributable to direct membrane damage. However, because compound-only interference controls containing MNQ and the corresponding fluorescent probes in the absence of bacterial cells were not performed, direct optical or probe interference cannot be completely excluded. Therefore, the fluorescence-based findings should be interpreted cautiously as being consistent with changes in ROS-associated signals, efflux activity, and membrane permeability rather than as definitive evidence of these mechanisms. Nevertheless, the combined ROS, EtBr accumulation, NPN, and PI results support the possibility that MNQ primarily modulates bacterial physiological processes rather than acting as a conventional membrane-disrupting antibacterial agent.

Beyond EPS composition, bacterial motility represents another critical determinant of biofilm formation and structural development [36]. Consequently, impairing motility has emerged as an alternative strategy for biofilm interference. *P. aeruginosa* possesses at least three distinct modes of motility: flagellum-dependent swimming, type IV pili (T4P)-mediated twitching on surfaces, and swarming across semi-solid surfaces, which involves coordinated flagellar activity and the production of biosurfactants such as rhamnolipids [2]. In our study, MNQ inhibited twitching and swimming motility of PA14 at 31.25–125 μg/mL, with motility showing a decreasing trend with increasing concentrations. In contrast, MNQ enhanced swarming motility, with the strongest effect observed at 62.5 μg/mL. This differential susceptibility suggests that the regulatory networks controlling the three motility types are at least partially independent in *P. aeruginosa*, reflecting the complexity of its behavioural adaptation. Swimming is primarily associated with flagellar mediated movement in liquid environments, whereas twitching depends predominantly on T4P-mediated surface translocation [37]. The simultaneous inhibition of swimming and twitching by MNQ may therefore reflect interference with multiple motility-associated processes, although the underlying molecular mechanisms remain to be elucidated. Given that swimming and T4P-mediated twitching contribute to surface exploration, attachment, and early biofilm development, their inhibition may contribute to the reduced biofilm formation observed following MNQ treatment. Collectively, these findings suggest that MNQ affects multiple biofilm-associated phenotypes, including EPS production, pyocyanin production, and motility. The quorum sensing (QS) network represents a central regulatory hub in *P. aeruginosa*, governing approximately 10% of its genome and orchestrating a wide array of virulence-associated phenotypes in a cell density-dependent manner [1]. This QS circuitry comprises four interconnected signalling systems: *lasR*/*lasI*, *rhlR*/*rhlI*, PQS, and IQS, which collectively control the production of proteases, rhamnolipids, toxins, hydrogen cyanide, siderophores, exopolysaccharides, pyocyanin, efflux pumps, motility, and biofilm formation [9]. Among these, the *las* system has historically been recognised as critical for biofilm development; however, subsequent studies have demonstrated that this effect is highly contingent upon growth conditions, underscoring the regulatory adaptability of the QS network [38,39]. In both PAO1 and PA14 strains, these three major QS systems (*las*, *rhl*, and PQS) are organised in a hierarchical manner. The *las* system, positioned at the apex of the regulatory cascade, activates both the *rhl* and PQS systems, whereas the *rhl* system negatively modulates PQS expression. In turn, the PQS system positively regulates the rhl circuit, highlighting the interconnectedness of the three QS systems.

Quantitative real-time PCR analysis showed that MNQ selectively affected the QS network of *P. aeruginosa*, with stronger suppression of the *las*/*rhl* systems and moderate effects on the PQS system. Similar transcriptional modulation of QS-associated genes has been reported for other small molecule compounds. For example, taxifolin and naringenin have been shown to downregulate QS-related genes, including *lasI*, *lasR*, *rhlI*, and *rhlR*, in *P. aeruginosa* PAO1 [40]. Quercetin has also been reported to interfere with *lasR*-mediated QS signalling, thereby reducing QS-regulated phenotypes such as biofilm formation, pyocyanin production, protease and elastase activity, and motility [41]. Moreover, sotolon was shown to suppress the expression of *lasI*, *lasR*, *rhlI*, and *rhlR* and to protect mice against *P. aeruginosa* pathogenesis [42]. Together, these findings suggest that small-molecule modulation of QS networks can attenuate multiple virulence-associated phenotypes independently of direct bactericidal activity. Notably, *rhlA* showed a stronger suppression than its upstream regulatory genes. This preferential response is consistent with previous observations for cinnamoyl hydroxamic acid (CHA) and 3-methoxycinnamoyl hydroxamic acid (MCHA), for which *rhlA* exhibited the strongest inhibition among the QS-associated genes examined in *P. aeruginosa* PAO1 [43]. The pronounced downregulation of *rhlA* may contribute to the reduced QS-associated phenotypes observed following MNQ treatment. Given the established role of *rhlA* as a downstream effector of the *rhl* system, its selective suppression may represent an important component of the antivirulence activity of MNQ. The consistent sensitivity of *rhlA* to these structurally distinct compounds further suggests that this gene may be particularly responsive to chemical perturbation of the QS regulatory network. However, whether the preferential regulation of *rhlA* results from direct interference with QS signalling or from indirect effects on downstream regulatory and metabolic processes remains to be determined [43]. In contrast to the suppression of QS-associated virulence genes, MNQ treatment significantly upregulated the biofilm dispersal-associated genes *dipA* and *bdlA*. The simultaneous activation of dispersal-associated pathways and suppression of QS-related genes may reflect a cellular stress response or a compensatory regulatory mechanism. When QS-mediated sessility and virulence programmes are perturbed, bacteria may activate dispersal-related pathways to facilitate a transition toward a more motile state. The increased expression of *dipA* and *bdlA* is also consistent with a possible alteration of intracellular c-di-GMP signalling, as these genes are associated with the regulation of biofilm dispersal. However, intracellular c-di-GMP concentrations were not directly measured in the present study. Therefore, whether MNQ promotes biofilm dispersal through modulation of c-di-GMP signalling requires further experimental verification. Collectively, these findings indicate that MNQ affects multiple regulatory processes in *P. aeruginosa*, including QS-associated pathways, downstream virulence-associated genes, and biofilm dispersal-related pathways. The pronounced reduction in *rhlA* expression further identifies this downstream effector as an important component of the MNQ-mediated antivirulence response and suggests that *rhlA* may represent a potential therapeutic target for attenuating *P. aeruginosa* virulence. Nevertheless, whether targeting *rhlA* or related downstream effectors can reduce the development of adaptive resistance requires further investigation.

In addition to its antivirulence properties, we further assessed the direct antibacterial activity of MNQ against PA14. Growth and metabolic inhibition assays revealed a decreasing trend in bacterial growth and metabolic activity with increasing MNQ concentrations; however, this inhibition remained modest even at the highest concentration tested, consistent with its relatively weak intrinsic antibacterial activity and high MIC (>500 μg/mL). This finding is in line with previous reports that naturally occurring quinone compounds can exert antimicrobial effects while also perturbing bacterial metabolic processes. For example, pyrroloquinoline quinone, a naturally occurring quinone compound, has been reported to exhibit antibacterial activity against *Staphylococcus aureus* and *Proteus vulgaris*, while also affecting bacterial metabolic activity, supporting the potential of quinone-based compounds as antimicrobial agents [44]. Natural compounds have also been investigated as antivirulence and QS inhibitors. Methyl gallate, exhibited pronounced QS inhibitory activity against *P. aeruginosa* PAO1 and PA14 at 1/4 MIC without substantially affecting bacterial viability [45]. Together with these previous findings, our results suggest that MNQ may exert its biological effects primarily through modulation of virulence-associated processes rather than direct inhibition of bacterial growth. Indeed, most natural quinones exhibit only mild or negligible direct antimicrobial action, suggesting that MNQ may primarily function as an adjuvant rather than a conventional antibiotic.

To test this hypothesis, we performed combination assays with clinically relevant antibiotics. At a concentration of 125 μg/mL, MNQ demonstrated synergistic or additive antibacterial effects when combined with azithromycin, cefepime, and ciprofloxacin. In contrast, no such synergy was observed with polymyxin B or tetracycline. In fact, the lack of synergistic effect between MNQ and polymyxin B was clearly demonstrated in Table 1. Given that increasing outer membrane permeability is one of the primary antibacterial mechanisms of polymyxin B [25], our finding that MNQ reduced outer membrane permeability compared with the control group may help explain the lack of synergistic interaction between MNQ and polymyxin B. This contrasting effect suggests that MNQ may interfere with membrane-related processes targeted by polymyxin B, potentially contributing to their non-synergistic interaction. Similarly, MNQ did not enhance the susceptibility of PA14 to tetracycline in our study. In contrast, Xu et al. reported a synergistic antibacterial effect between tetracycline and MNQ, particularly against *Escherichia coli* carrying Tet(X3)/Tet(X4) [31]. This antibiotic-selective pattern implies that MNQ’s adjuvant activity is not universally applicable but rather depends on the specific antibiotic class, potentially reflecting differences in bacterial uptake, target accessibility, or the underlying mechanisms of action. The mechanistic basis of this selectivity warrants further investigation, as it may inform optimal combination strategies for future therapeutic applications.

Despite the promising findings presented here, several limitations of this study should be acknowledged. First, our antibiofilm characterisation was confined to the laboratory strain *P. aeruginosa* PA14, and the effects of MNQ on biofilms formed by clinical isolates, particularly multidrug-resistant strains, and other clinically relevant pathogens remain to be determined. Second, the present study did not include cytotoxicity or safety assessments in relevant mammalian cells; therefore, the selectivity and safety profile of MNQ remain to be established. Third, the characterisation of biofilm architecture was limited, and more comprehensive imaging approaches, such as high-resolution confocal microscopy and three-dimensional analysis, would provide further insights into the effects of MNQ on biofilm structure and viability. Finally, all experiments were performed in vitro, and the efficacy and safety of MNQ have not yet been validated in animal models. Future in vivo studies are therefore necessary to evaluate its therapeutic potential and safety under physiologically relevant conditions. In addition, although MNQ impaired swimming and twitching motility but not swarming motility, the molecular basis underlying this differential susceptibility remains unresolved and warrants further investigation.

## 5. Conclusions

In summary, MNQ exhibits a multifaceted antibiofilm and antivirulence profile against *P. aeruginosa* PA14, acting through concurrent modulation of EPS composition, motility, antioxidant ability, QS signalling, and antibiotic adjuvant activity. While MNQ’s modest intrinsic antibacterial effect suggests it is better suited as a synergistic partner rather than a stand-alone antimicrobial, its ability to potentiate multiple clinically relevant antibiotics combined with its natural origin and pleiotropic mode of action renders it an attractive scaffold for further optimisation. Future investigations should prioritise mechanistic dissection of its target specificity, evaluation against clinical isolates, and in vivo efficacy assessment to fully realise its therapeutic promise.

## Figures and Tables

**Figure 1:**
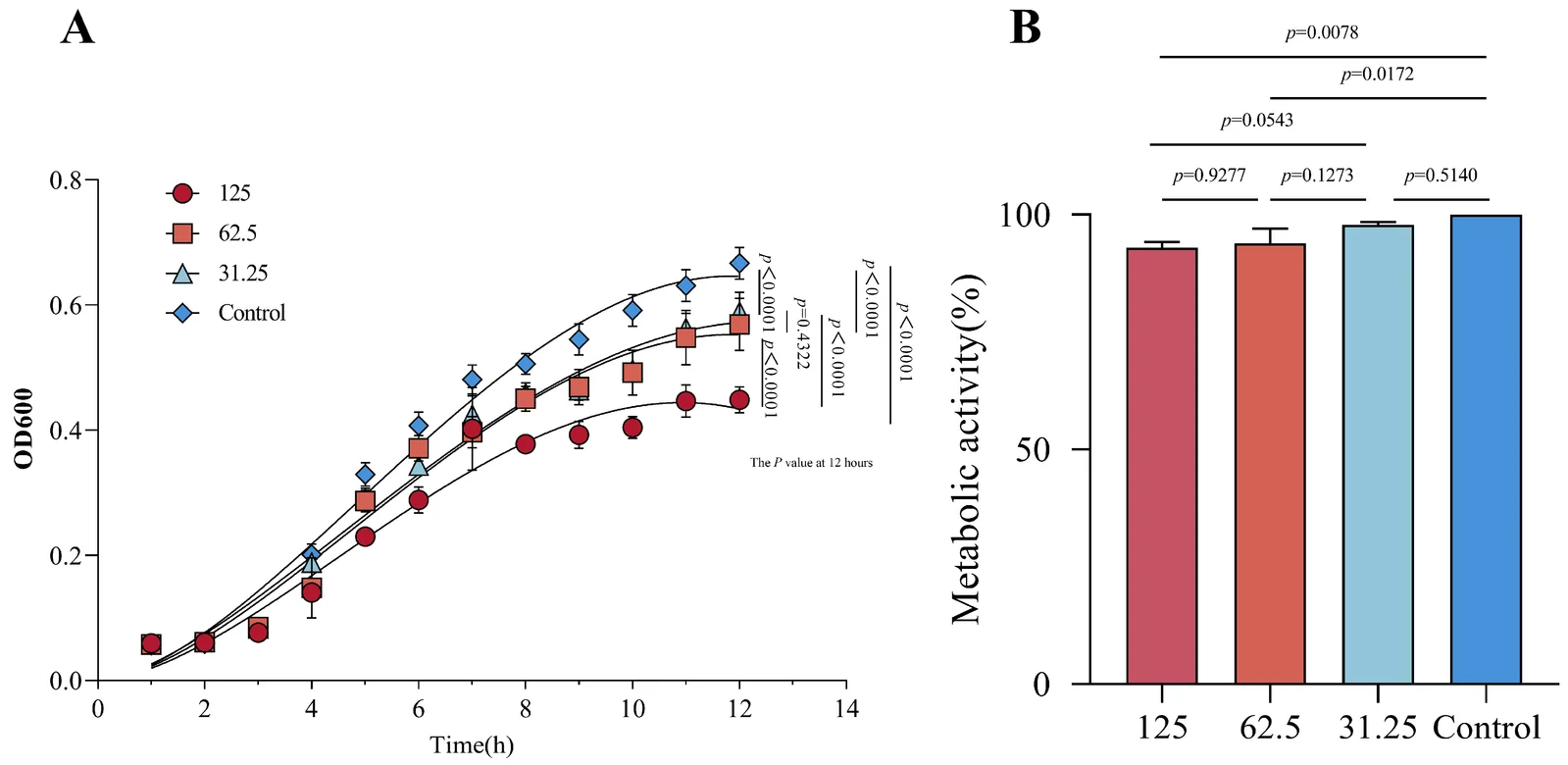
Effects of MNQ on the growth and metabolic activity of *P. aeruginosa* PA14. (**A**) Growth curves of PA14 treated with different concentrations of MNQ (31.25, 62.5, and 125 μg/mL) over 12 h. (**B**) Metabolic activity of PA14 after treatment with different concentrations of MNQ, expressed as a percentage of the untreated control. Statistical significance was determined by two-way ANOVA followed by Tukey’s multiple comparisons test (**A**) and one-way ANOVA followed by Tukey’s multiple comparisons test (**B**). Data are presented as means ± SD. *p* < 0.05 was considered statistically significant.

**Figure 2:**
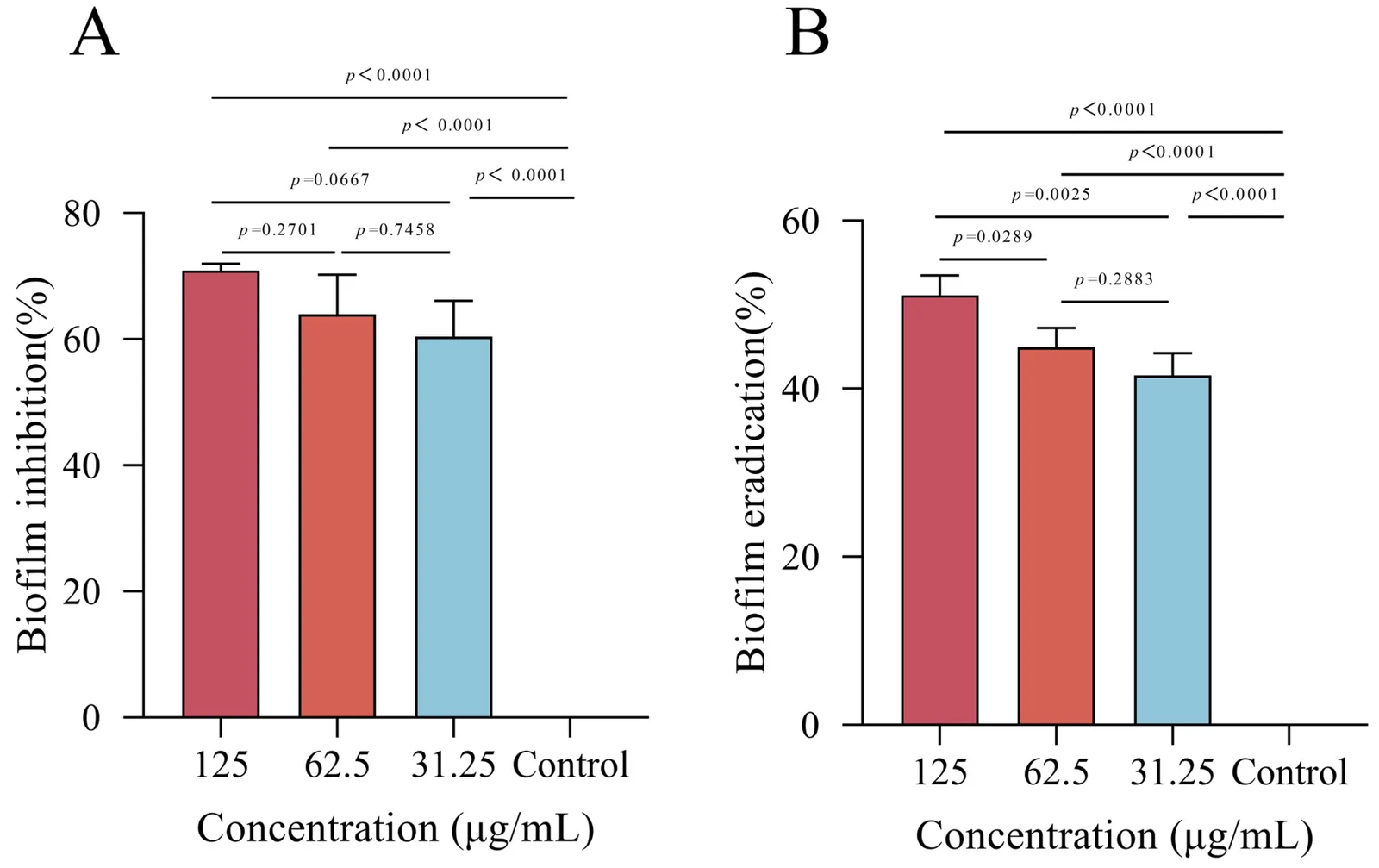
MNQ inhibits biofilm formation (**A**) and eradicates pre-existing biofilms (**B**) at the tested concentrations. Statistical significance was determined by one-way ANOVA followed by Tukey’s multiple comparisons test. Data are presented as mean ± SD. *p* < 0.05 was considered statistically significant.

**Figure 3:**
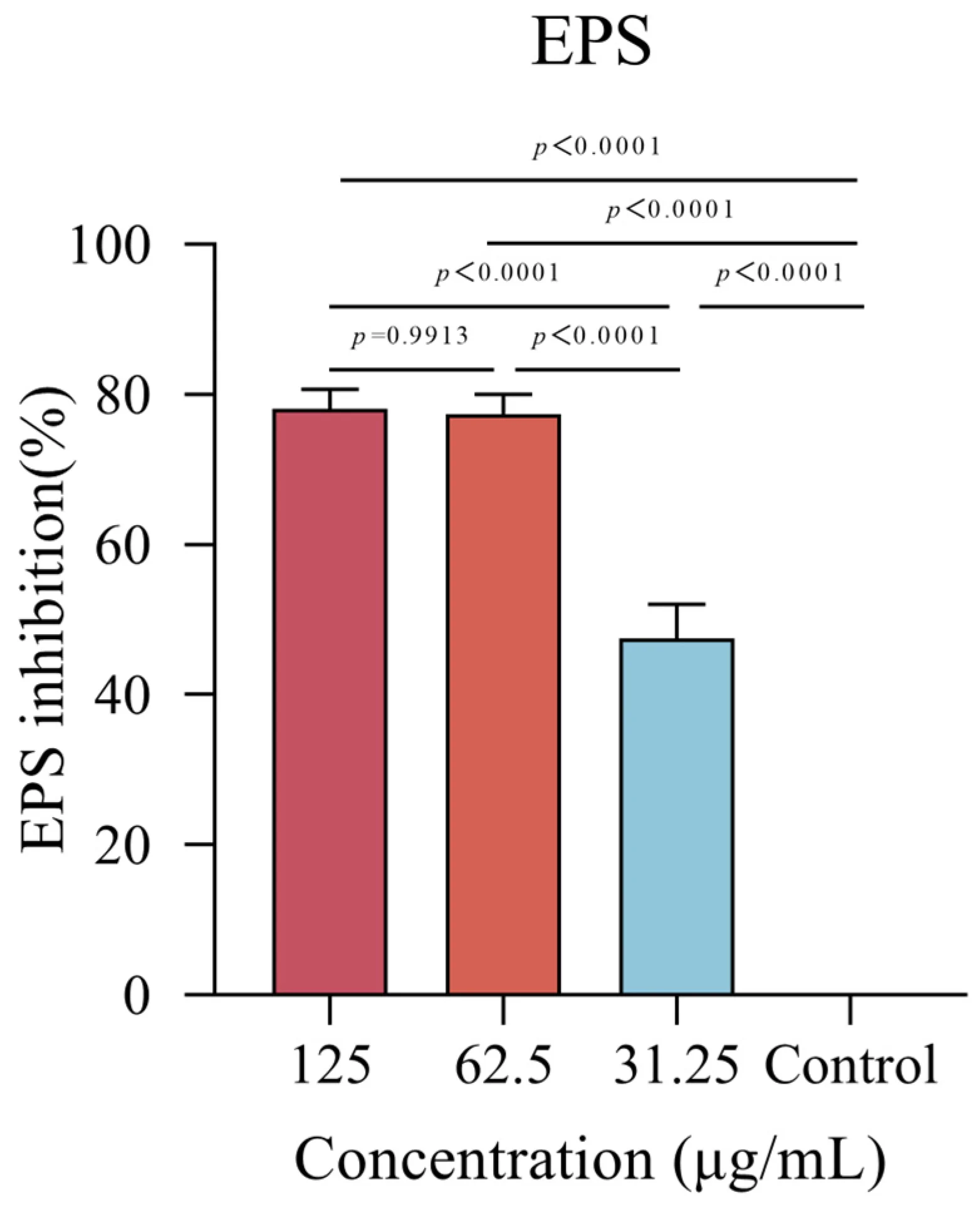
MNQ significantly reduces the production of extracellular polymeric substances (EPS) at the tested concentrations. Statistical significance was determined by one-way ANOVA followed by Tukey’s multiple comparisons test. Data are presented as mean ± SD. *p* < 0.05 was considered statistically significant.

**Figure 4:**
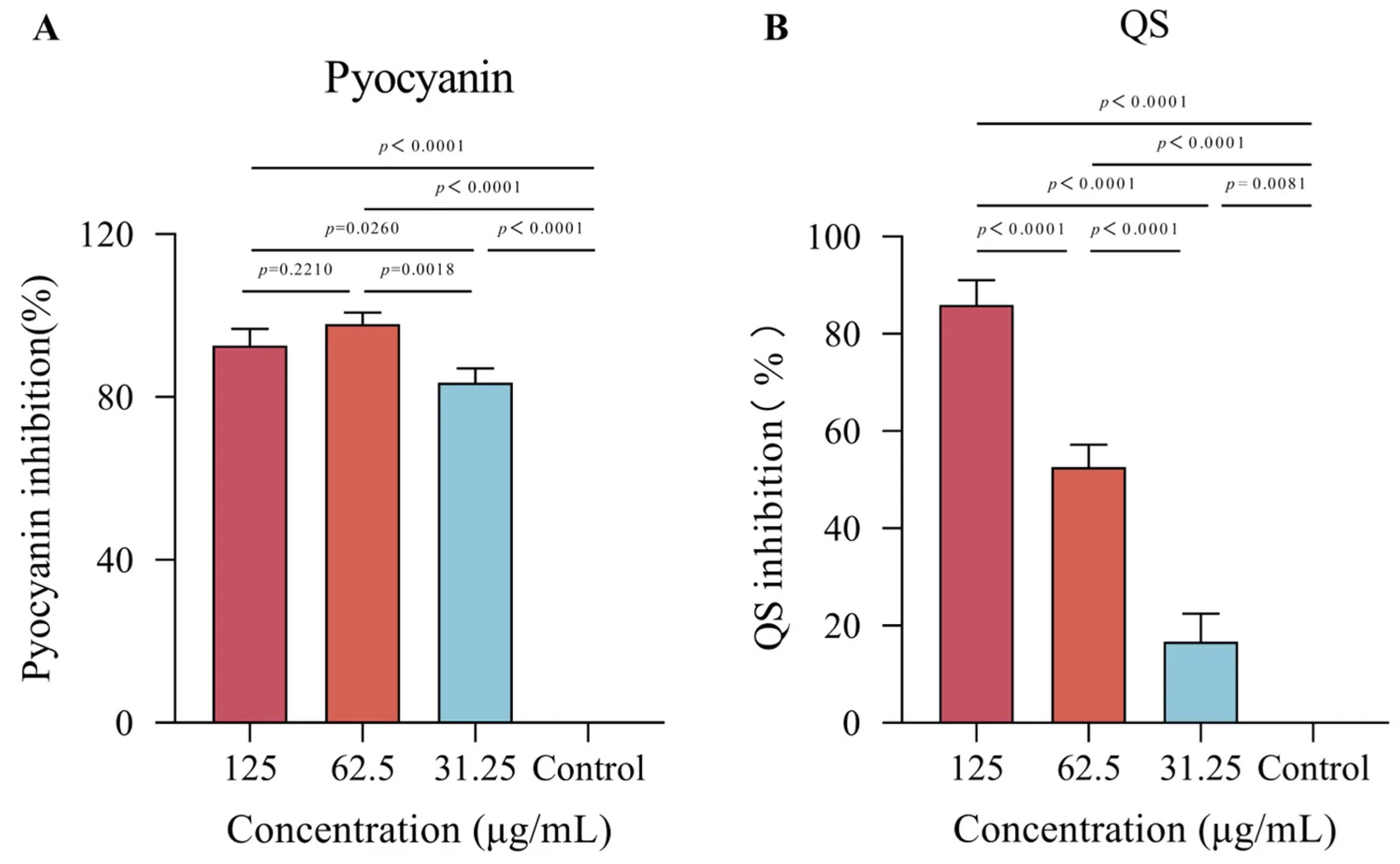
Inhibitory effects of MNQ on pyocyanin production (**A**) and quorum sensing (QS) activity (**B**). Different concentrations of MNQ (125, 62.5, and 31.25 μg/mL) were applied to evaluate the inhibitory percentage. Statistical significance was determined by one-way ANOVA followed by Tukey’s multiple comparisons test. Data are presented as mean ± SD. *p* < 0.05 was considered statistically significant.

**Figure 5:**
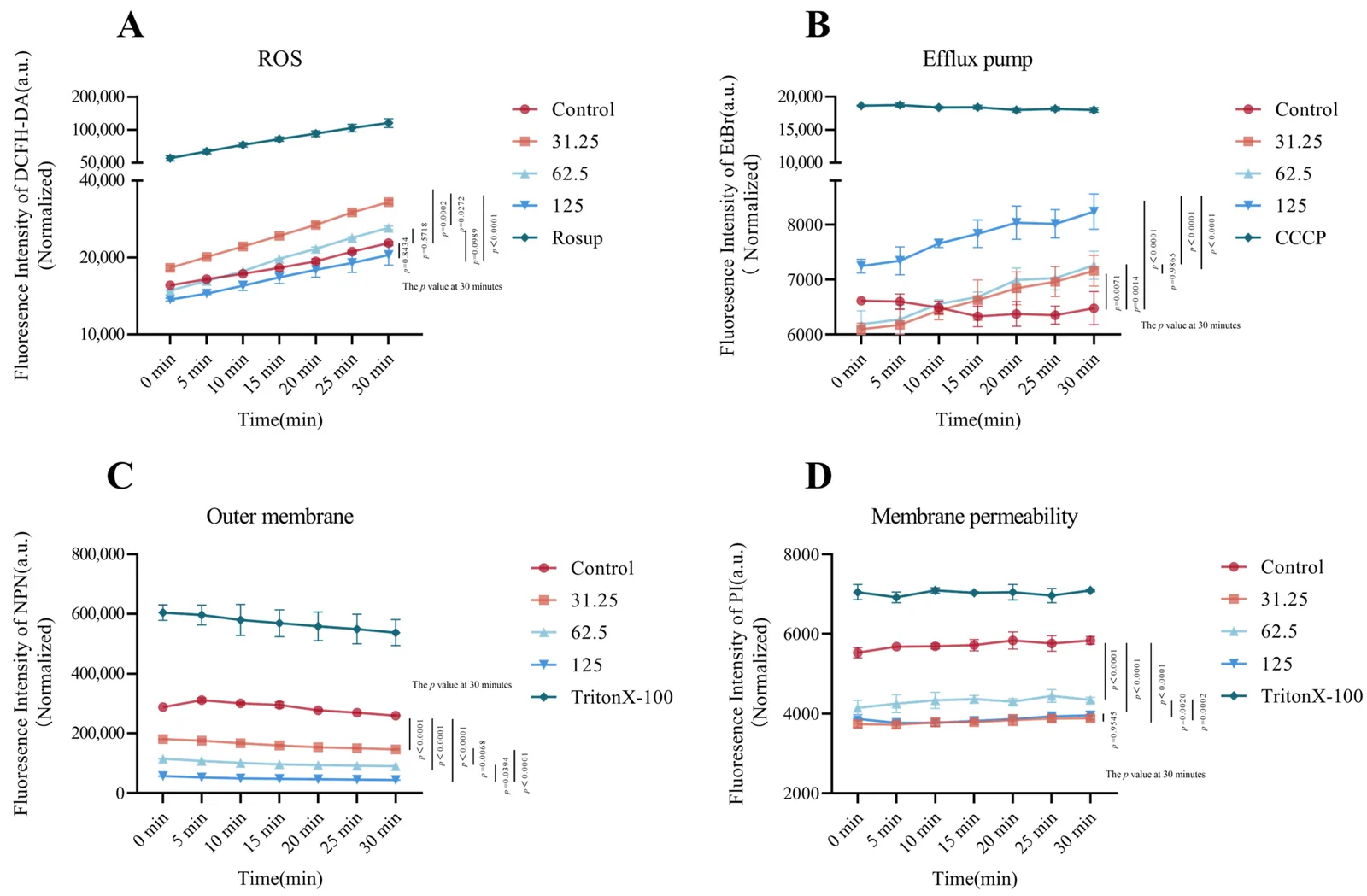
Effects of MNQ on intracellular ROS accumulation, efflux pump activity, and membrane integrity in *P. aeruginosa* PA14. Bacteria were treated with varying concentrations of MNQ (31.25, 62.5, and 125 μg/mL) and assessed using fluorescence probes. (**A**) Intracellular ROS levels measured by DCFH-DA fluorescence; Rosup was used as a positive control. (**B**) Efflux pump activity was evaluated by EtBr accumulation; CCCP (a protonophore) was used as a positive control for efflux pump inhibition, showing increased EtBr fluorescence. (**C**) Outer membrane permeability was assessed by NPN staining. TritonX-100 was used as a positive control. (**D**) Inner membrane permeability assessed by PI staining. TritonX-100 was used as a positive control. Statistical significance was determined by two-way ANOVA followed by Tukey’s multiple comparisons test. Data are presented as mean ± SD. *p* < 0.05 was considered statistically significant.

**Figure 6:**
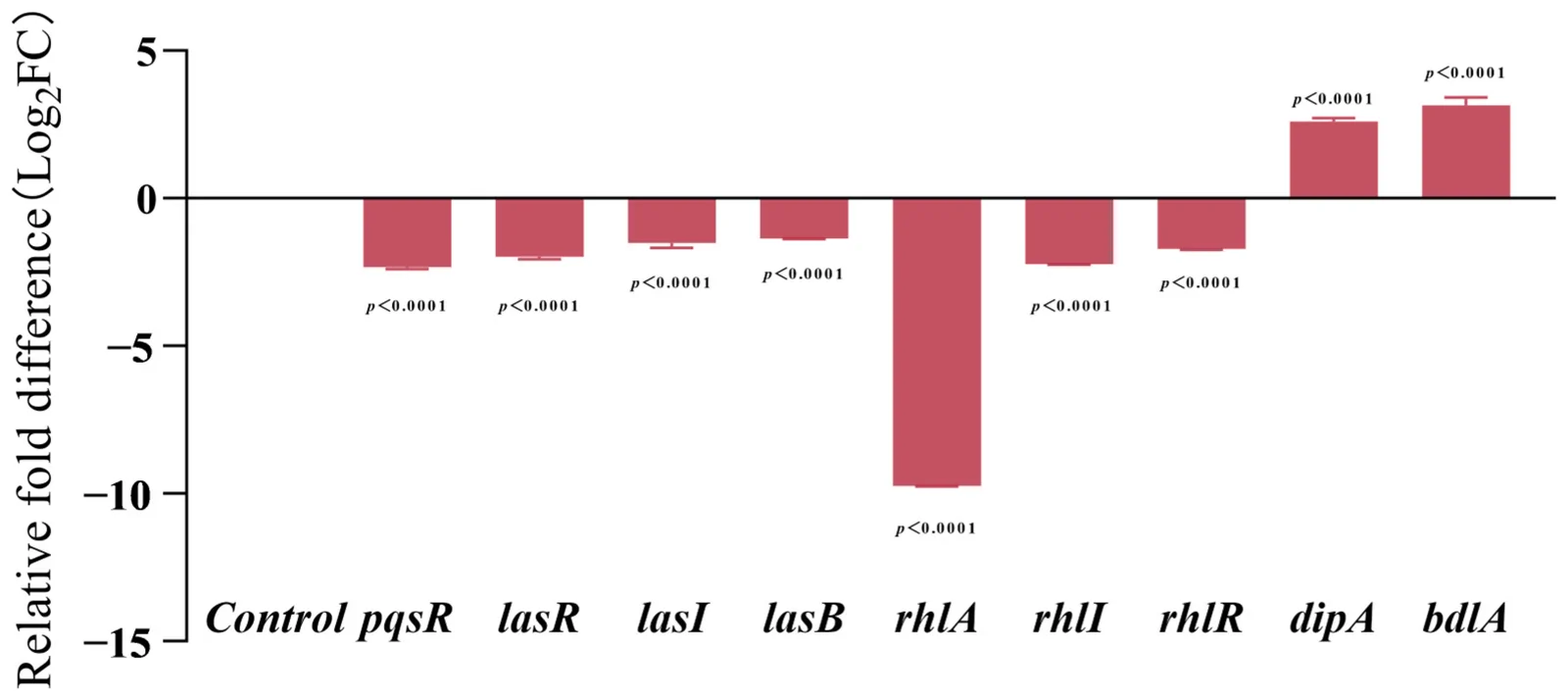
Effects of MNQ on transcription of biofilm-related genes. qRT-PCR analysis revealed significant transcriptional alterations in nine key genes (*pqsR*, *lasR*, *lasI*, *lasB*, *rhlA*, *rhlI*, *rhlR*, *dipA*, and *bdlA*) compared to the control group. Statistical significance was determined by one-way ANOVA followed by Dunnett’s multiple comparisons test. Data are presented as mean ± SD. *p* < 0.0001 compared with the control group.

**Figure 7:**
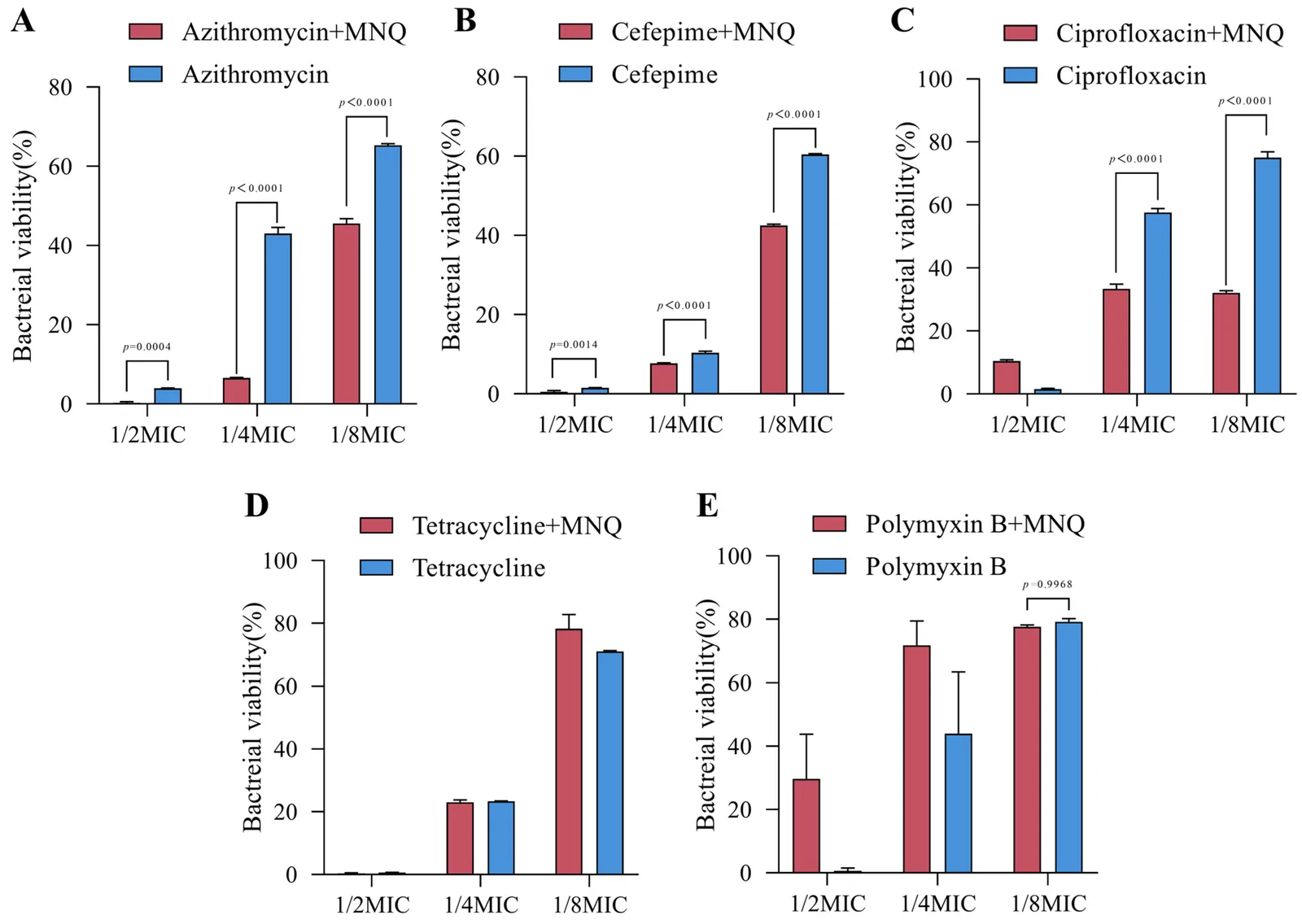
Effects of MNQ (125 μg/mL) combined with antibiotics on bacterial viability of PA14. (**A**) Azithromycin. (**B**) Cefepime. (**C**) Ciprofloxacin. (**D**) Tetracycline. (**E**) Polymyxin B. Statistical significance was determined by two-way ANOVA followed by Šídák’s multiple comparisons test. Data are presented as mean ± SD. *p* < 0.05 was considered statistically significant.

**Table 1:** Minimum inhibitory concentrations (MICs) and fractional inhibitory concentration indices (FICIs) of antibiotics in combination with MNQ against *P. aeruginosa* PA14.

Drugs	Alone (μg/mL)	With MNQ (125 μg/mL)	FICI	Interpretation
Azithromycin	125	31.250	0.250 < x < 0.500	synergistic
Cefepime	3.900	0.977	0.250 < x < 0.258	synergistic
Ciprofloxacin	0.122	0.061	0.500 < x < 0.504	additive
Tetracycline	15.625	15.625	>1	indifferent
Polymyxin B	3.900	3.900	>1	indifferent
MNQ	>500	-	-	-

Note: FICI values are reported as ranges or upper limits because the MIC of MNQ against *P. aeruginosa* PA14 was >500 μg/mL, exceeding the highest concentration tested and therefore could not be determined as an exact numerical value. The FICI values were calculated using the experimentally determined MIC endpoints. FICI ≤ 0.5, 0.5 < FICI ≤ 1.0, and FICI > 1.0 indicate synergistic, additive, and indifferent interactions, respectively.

## Data Availability

The original contributions presented in this study are included in the article/Supplementary Material. Further inquiries can be directed to the corresponding authors.

## Supplementary Materials

The following supporting information can be downloaded at: https://www.mdpi.com/article/10.3390/biom16101394/s1, Figure S1: Motility inhibition assays of *P. aeruginosa* PA14; Table S1: Primer sequence information.
