# Supplementary material for: Naturally Occurring 2-Methoxy-1,4-naphthoquinone Attenuates Biofilm Formation and Quorum Sensing-Regulated Virulence in Pseudomonas aeruginosa
Source: Biomolecules. 2026 Sep 24;16(10):1394. doi: 10.3390/biom16101394 (PMC13702217; doi:10.3390/biom16101394)
Supplement: Supplementary file 1 [file biomolecules-16-01394-s001.zip › biomolecules-4489961-supplementary.pdf]

Supplementary Materials

Table S1 Primer sequence information

Gene name	Primer orientation	Sequence (5′-3′)
lasR	Forward	CATCGTCGCAACTACCC
	Reverse	GCGCACCACTGCAACACT
lasI	Forward	TTGCTCGCCGCACATC
	Reverse	GGCACGGATCATCATCTT
lasB	Forward	GTTCTATCCGCTGGTGTCG
	Reverse	CGCTGCCCTTCTTGATG
rhlI	Forward	ATCCGCAAACCCGCTAC
	Reverse	GCAGGCTGGACCAGAATAT
rhlR	Forward	TCGGAAATGGTGGTCTGG
	Reverse	CGCTCGAAGCTGGAGATG
rhlA	Forward	AACGAGACCGTCGGCAAAT
	Reverse	GCTCCAGGCAAGCCAAGTAG
bdlA	Forward	CGGAACGCACCAGCAAGTC
	Reverse	TGCACCGCTTCTACCACCTT
pqsR	Forward	AACATGTTCCTCCAGGTCAT
	Reverse	GTTGAGATTGAAGGCGATGT
dipA	Forward	TGCCATCTTGGGCGCAGTCGC
	Reverse	ATTAATGGCAGGCTGACTTGATGG

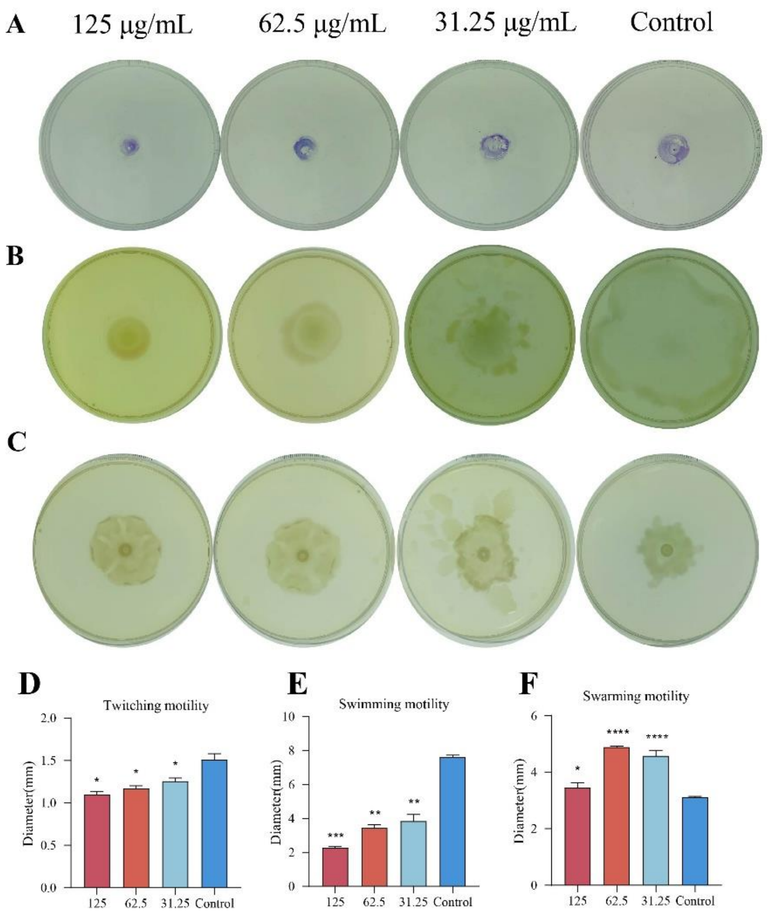

Figure S1. Motility inhibition assays of *P. aeruginosa* PA14. Three motility assays were conducted on plates containing MNQ on agar at different concentrations (125, 62.5, 31.25 μg/mL): (A) Twitching motility; (B) Swimming motility; (C) Swarming motility; (D) Diameter of the twitching motility zone; (E) Diameter of the swimming motility zone; (F) Diameter of the swarming motility zone. Statistical significance was determined by one-way ANOVA followed by Tukey's or Dunnett's multiple comparisons test, as appropriate. Data are presented as means ± SD. $P < 0.05$ was considered statistically significant.
